# Dendritic Spikes in Sensory Perception

**DOI:** 10.3389/fncel.2017.00029

**Published:** 2017-02-15

**Authors:** Satoshi Manita, Hiroyoshi Miyakawa, Kazuo Kitamura, Masanori Murayama

**Affiliations:** ^1^Laboratory for Behavioral Neurophysiology, Brain Science Institute, RIKENWako City, Saitama, Japan; ^2^Department of Neurophysiology, Division of Medicine, University of YamanashiChuo-shi, Japan; ^3^Laboratory of Cellular Neurobiology, Tokyo University of Pharmacy and Life SciencesHachioji, Japan

**Keywords:** dendritic spike, sensory perception, dendritic integration, pyramidal neuron, neocortex, top-down control, bottom-up processing

## Abstract

What is the function of dendritic spikes? One might argue that they provide conditions for neuronal plasticity or that they are essential for neural computation. However, despite a long history of dendritic research, the physiological relevance of dendritic spikes in brain function remains unknown. This could stem from the fact that most studies on dendrites have been performed *in vitro*. Fortunately, the emergence of novel techniques such as improved two-photon microscopy, genetically encoded calcium indicators (GECIs), and optogenetic tools has provided the means for vital breakthroughs in *in vivo* dendritic research. These technologies enable the investigation of the functions of dendritic spikes in behaving animals, and thus, help uncover the causal relationship between dendritic spikes, and sensory information processing and synaptic plasticity. Understanding the roles of dendritic spikes in brain function would provide mechanistic insight into the relationship between the brain and the mind. In this review article, we summarize the results of studies on dendritic spikes from a historical perspective and discuss the recent advances in our understanding of the role of dendritic spikes in sensory perception.

## A Historical Perspective of Dendritic Spike Studies

Early *in vivo* studies in the 1950s suggested that spikes evoked near the soma propagate into dendrites (Chang, 1951; Bishop and Clare, 1953; Fatt, 1957a,b; Eccles et al., 1958; Terzuolo and Araki, 1961). Results of some studies also suggested that spikes are locally initiated at dendrites before the generation of somatic action potentials (Cragg and Hamlyn, 1955; Andersen, 1960; Spencer and Kandel, 1961; Fujita and Sakata, 1962). These ideas were supported by intracellular sharp electrode recordings from the dendrites of several types of neurons (Cragg and Hamlyn, 1955; Andersen, 1960; Fujita and Sakata, 1962; Llinás and Nicholson, 1971; Wong et al., 1979). Subsequent studies also investigated the electrical properties of dendrites by performing whole-cell recordings in brain slices (Stuart et al., 1993; Stuart and Sakmann, 1994; Spruston et al., 1995; Magee and Johnston, 1997). These studies demonstrated that backward-propagating spikes (backpropagating action potential, BPAP) from the soma are Na^+^ spikes. Studies conducted more recently showed that Na^+^ spikes can be locally initiated in dendrites in response to synaptic inputs, before the initiation of somatic spikes (Kim and Connors, 1993; Stuart et al., 1997; Golding and Spruston, 1998; Ariav et al., 2003; Gasparini et al., 2004; Gasparini and Magee, 2006; Losonczy and Magee, 2006; Nevian et al., 2007). *In vitro* studies identified dendritic spikes in various types of neurons, and showed that these dendritic spikes are mediated by voltage-gated Ca^2+^ channels (Ca^2+^ spikes) or *N-methyl-*D*-aspartate* (NMDA) receptor channels (NMDA spikes; see reviews, Larkum and Nevian, 2008; Antic et al., 2010; Major et al., 2013). Taken together, these extensive *in vitro* studies have revealed unique electrical properties of dendrites, either passive or active, that enable neurons to output spikes by integrating synaptic inputs in various ways. These studies stimulated dendritic research aimed at understanding the physiological functions of dendritic spikes.

Why do dendrites have spikes? It is reasonable to assume that dendritic spikes have physiological functions for neuronal computation and synaptic plasticity in single neurons and circuits (Poirazi and Mel, 2000; London and Häusser, 2005). Dendritic Ca^2+^ spikes can trigger somatic repetitive action potentials (Connors and Gutnick, 1990; Amitai et al., 1993; Kim and Connors, 1993; Schiller et al., 1997; Helmchen et al., 1999; Larkum et al., 1999; Larkum and Zhu, 2002). Multiple NMDA spikes trigger a dendritic Ca^2+^ spike, which can induce somatic burst firing; however, a single NMDA spike cannot trigger a somatic action potential or can trigger only a single action potential (Milojkovic et al., 2004; Polsky et al., 2004, 2009; Larkum et al., 2009; Lavzin et al., 2012; Palmer et al., 2014). These dendritic spikes increase intracellular concentrations of Ca^2+^, which acts as a second messenger in cells and induces synaptic plasticity (note that plasticity such as long-term potentiation that does not depend on dendritic spikes has also been reported, Dudman et al., 2007), resulting in modification of somatic firings (Golding et al., 2002; Holthoff et al., 2006; Kampa et al., 2007; Johnston and Narayanan, 2008; Losonczy et al., 2008; Sjöström et al., 2008; Spruston, 2008; Takahashi and Magee, 2009; Kim et al., 2015; Basu et al., 2016). Because somatic spikes have been considered as a basic unit of information in the brain, dendritic spikes likely play an important role in information processing in the brain. In addition, the occurrence of dendritic spikes is controlled by various mechanisms such as spatiotemporal patterns of synaptic inputs (Gasparini et al., 2004; Gasparini and Magee, 2006; Losonczy and Magee, 2006; Branco and Häusser, 2010) and inhibitory synaptic inputs (Pérez-Garci et al., 2006; Murayama et al., 2009; Palmer et al., 2012). Therefore, dendritic spikes should increase the computational power of the system.

Several aspects of dendritic spikes, such as the cellular mechanisms underlying their generation and their contribution to computation in a single neuron and neural circuits, have been extensively investigated. However, little is known about the functional relevance of dendritic spikes in perception, which was one of the original and fundamental questions raised 50 years ago, when the existence of dendritic spikes was proposed (Spencer and Kandel, 1961; Llinás et al., 1968). In the last two decades, technical advances, such as two-photon microscopy, genetically encoded Ca^2+^ indicators (GECIs), and transgenic animals, have enabled researchers to examine dendritic activities in conjunction with animal behavior. Furthermore, neural activities can be manipulated using optogenetic methods with high spatiotemporal resolution. Emerging lines of evidence generated using these state-of-the-art technologies have shown the physiological importance of dendritic spikes. Comprehensive reviews of dendritic mechanisms for synaptic integration and plasticity *in vivo* have been published recently (Grienberger et al., 2015; Stuart and Spruston, 2015; Palmer et al., 2016). In this review article, we summarize recent advances in elucidating not only the relationship between dendritic spikes and perception but also their causal relationship, focusing on Ca^2+^ and NMDA spikes in pyramidal neurons.

## Are Dendritic Spikes Necessary for Perception?

### Ca^2+^ Spikes

**Figure 1:**
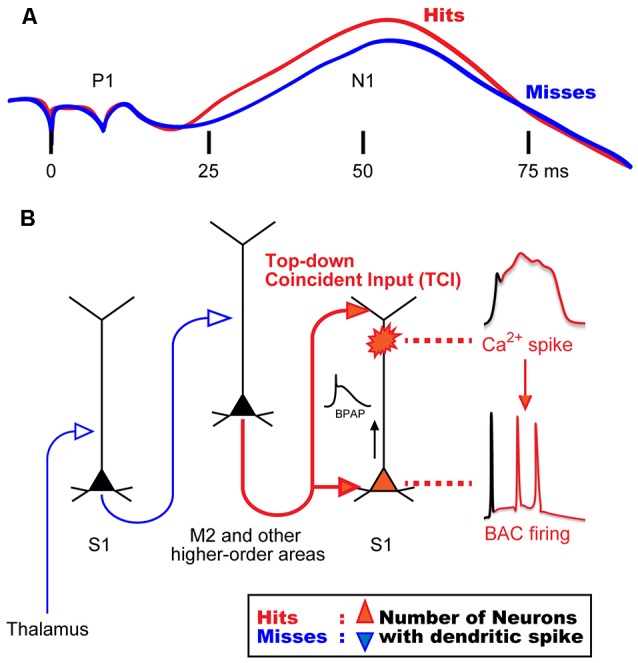
**Hypothetical model for mechanisms of sensory perception. (A)** Animal perception, assumed by behavioral responses (i.e., hit or miss behaviors), correlates with the late component in a stimulus intensity discrimination task. Even though the intensity of sensory stimulation was identical, animal behavior responses were different and perceptual behaviors correlated with the late neural activities. **(B)** A novel mechanism for the late activity. Top-down inputs (here from M2) propagate from the bottom to the top layers of the sensory cortex in a short time window to reliably drive dendritic spiking and BAC firing (~50 ms in **A**). We termed this top-down input pattern, “top-down coincident input or TCI”. We hypothesized that the number of neurons that had a Ca^2+^ spike during perception (Hits) behavior is larger than that during no perception (Misses). BAC, backpropagating action-potential-activated calcium spike; BPAP, backpropagating action potential. Panel **(A)** is reprinted with permission from Cauller (1995).

Previous studies in awake monkeys revealed that somatosensory stimulation induces a surface potential on electroencephalogram (EEG), termed “somatosensory evoked potential (SEP)”, which represents the responses of a population of neurons in the sensory cortex (Cauller and Kulics, 1991). SEP consists of early and late components: a primary surface-positive component (P1) and a secondary surface-negative component (N1). The early component correlates with external information such as stimulus intensity, whereas the late component represents behaviors that require perception of the stimulation (Figure 1A; Kulics et al., 1977; Kulics, 1982; Cauller and Kulics, 1988, 1991; Cauller, 1995). The late components evoked by sensory stimulation have also been observed in dendrites during Ca^2+^ imaging of the rat S1 (Murayama and Larkum, 2009), and in other sensory areas of mammals via different recording techniques such as EEG, local field potential (LFP), and unit-recordings. These late components appear to be involved in perception (Lamme, 2001; Supèr et al., 2001; Del Cul et al., 2007; Meyer, 2011). Cauller (1995) hypothesized that the late component is mediated by dendritic spikes evoked by top-down inputs from higher-order brain areas such as the prefrontal cortex to the sensory area. However, no direct evidence has been obtained to prove this hypothesis. We recently investigated neural mechanisms underlying the late component of SEP, and reported that dendritic Ca^2+^ spikes in layer (L) 5 pyramidal neurons in the somatosensory cortex are involved in evoking this late component (Manita et al., 2015).

In our previous study (Manita et al., 2015), we used multi-unit recording (MUR) and LFP recording to measure neural activities evoked by hindpaw stimulation in the primary somatosensory cortex (S1) of mice. We identified early and late components similar to the two components of SEP in monkeys (Kulics et al., 1977; Kulics, 1982; Cauller and Kulics, 1988, 1991; Cauller, 1995). We showed that the early component is evoked by sensory inputs from the thalamus carrying information of hindpaw stimulation, and that this component activates the secondary motor cortex (M2), which is one of the higher-order brain areas. The late component was due to “top-down” feedback input from M2. Our viral tracing studies revealed that M2 neurons send their axons back to the S1 area, thus forming a reverberating circuit. The axons from M2 form synaptic contacts with both apical and basal dendrites of L5 pyramidal cells in S1 (Figure 1B). While the late component of the LFP is considered to be due to synaptic inputs from M2, the late component of MUR represents burst firing of somatic fast Na^+^ spikes that are sustained for a short period. Therefore, a mechanism must exist by which the synaptic inputs from M2 to the apical and basal dendrites give rise to burst firing at the soma.

Previous *in vitro* and simulation studies showed that the dendritic Ca^2+^ spike in L5 pyramidal neurons is induced by pairing of subthreshold depolarizations of distal apical dendrites and BPAPs, which can be evoked by synaptic inputs to tuft and proximal dendrites, respectively (Larkum et al., 1999; Xu et al., 2012; Larkum, 2013; Shai et al., 2015). Larkum et al. (1999) named these dendritic Ca^2+^ spikes as backpropagating action-potential-activated calcium spikes (BACs), and the resulting somatic burst firings as BAC firings.

In our study (Manita et al., 2015), current source density analysis of LFP using a linear probe showed that M2 top-down inputs coincidentally arrive at deep and superficial layers in S1 where basal and distal dendrites of L5 neurons located, respectively. By using two-photon microscopy and GECI, G-CaMP7 (Sato et al., 2015), we showed that M2 inputs evoke BACs in the dendrites of L5 neurons in the S1. Next, we expressed archaerhodopsin (ArchT; Han et al., 2011) in M2 neurons and optically inhibited axon terminals of M2 neurons extending to S1. The inhibition of the top-down signal from M2 to S1 decreased somatic firings of L5 neurons in the S1 during the late component. We hypothesized that the firing was due to BACs because pharmacological inactivation of M2 activity resulted in blockade of dendritic Ca^2+^ spikes and somatic firing. Furthermore, optical inhibition of this top-down signal impaired the ability of mice to discriminate surface textures (e.g., smooth and rough textures). Taken together, these results suggest that dendritic spikes are critical and necessary for sensory perception.

Supporting this idea, a recent study showed that the appearance of dendritic Ca^2+^ spikes in L5 pyramidal neurons of S1 is correlated with perception in mice (Takahashi et al., 2016). In that study, dendritic Ca^2+^ spikes were measured during a perceptual detection task with whisker stimulation. The authors showed that a group of dendritic Ca^2+^ spikes and the resulting somatic firings are related to “hit” trials (i.e., perception of whisker stimulation) in mouse behavior. To investigate whether the dendritic Ca^2+^ activity causes mice perceptual behavior, they inhibited or activated dendritic activity by using a pharmacological agent or optogenetic manipulations. The inhibition or activation of dendritic activity decreased or increased the detection probability of the mice, respectively. These results demonstrated that the dendritic Ca^2+^ spike is causally related to perceptual decision in mice. Although further investigation will be needed to support the hypothesis that dendritic spikes represent the neural correlates of perception, the experimental data of this study are consistent with this hypothesis.

Larkum (2013) proposed that BAC firing requires the cell to receive inputs from both bottom-up and top-down pathways, and that these inputs need to coincide within a narrow window of time. In our study (Manita et al., 2015), however, because the delay between the bottom-up input (early component) and the top-down input (late component) was too long, the coincidence would not occur. We hypothesize that the somatic burst firing during the late component is due to BAC in the dendrites, and that BAC is evoked by the coincidence of the two separate synaptic contacts onto the basal and apical dendrites sent from M2 alone, not due to the coincidence of M2 input with thalamic inputs. This mechanism is possible because the top-down projection from M2 to lower and upper layers in S1 allows M2 neurons alone to generate coincident inputs onto separate parts of the dendrites of S1 neurons. We have named this type of input pattern “top-down coincident input (TCI)” to directly define the role of top-down input in the generation of dendritic spikes. Regarding conventional top-down control system (Gilbert and Sigman, 2007; Gilbert and Burgess, 2008), sensory neurons may combine bottom-up and top-down inputs to induce BAC firing, as suggested by Larkum (2013).

The brain may utilize similar top-down inputs, that we described (Manita et al., 2015), from parts of the brains other than M2 to generate the BAC and hence the late components for perception. Moreover, the TCI *per se* would enable sensory perception without inputs from the other areas of the brain responsible for higher functions such as attention and motivation, or even without sensory information. TCI without external sensory inputs would be related to the phenomenon of hallucination (or illusion), a perceptual experience without sensory stimulation. A previous human study has shown that besides S1, premotor and prefrontal cortices are activated during illusory perception (Blankenburg et al., 2006). Some patients with non-convulsive status epilepticus of frontal origin have reported somatic hallucinations. Single-photon emission computed tomography (SPECT) during somatic hallucinations showed activation not only in the frontal area but also in the parietal area, including S1 as the projected regions (Takaya et al., 2005). In addition, Powers et al. (2016) discussed that hallucinations are involved in top-down processes. We hypothesize that, in some pathological states (e.g., epilepsy), TCI even without sensory input causes BAC firing, resulting in somatic hallucination.

Several input pathways to S1 are considered to be involved in the generation of dendritic Ca^2+^ spikes. Xu et al. (2012) reported that inputs from the vibrissal motor cortex (vM1) combined with thalamic inputs induce dendritic Ca^2+^ spikes in the barrel cortex during active touch by whiskers (i.e., whisking behavior), suggesting that spike generation requires both sensory and motor information. Such dendritic Ca^2+^ spikes induced by the combination of inputs from different pathways likely have different physiological roles from those of spikes induced by each input pathway. For example, the Ca^2+^ spikes that Xu et al. (2012) reported may carry motor information from the whiskers as well as information of touch perception.

### NMDA Spikes

Although it has been reported that induction of dendritic NMDA spikes require spatially and temporally clustered synaptic inputs, a previous simulation study has shown that *in vivo*-like background inputs lower the threshold for dendritic NMDA spikes without the clustered inputs and increase the efficacy of NMDA spikes to generate somatic spikes (Farinella et al., 2014). These results suggest that dendritic NMDA spikes have a greater impact on somatic spikes than that would be expected from the results of previous *in vitro* studies. Several studies have shown that whisker deflections induce NMDA-dependent depolarizations, presumably due to dendritic NMDA spikes in L 2/3 and 4 neurons, and that the spikes amplify thalamic inputs and determine somatic spiking (Lavzin et al., 2012; Gambino et al., 2014). In addition to whisker systems, studies on hindpaw somatosensory and visual systems have shown that dendritic NMDA spikes and consequent somatic spikes are induced by stimuli in L 2/3 neurons in mice (Smith et al., 2013; Palmer et al., 2014). Lavzin et al. (2012) and Smith et al. (2013) showed that NMDA spikes encode direction selectivity of whisker stimulation and orientation tuning of visual stimulation, respectively. Recently, it has been shown that orientation selective and spatially-clustered synaptic inputs correlate with the occurrence of dendritic Ca^2+^ events, which are presumably NMDA spikes in L 2/3 neurons of the visual cortex (Wilson et al., 2016), suggesting that the orientation tuning of somatic spikes are strongly influenced by that of dendritic spikes. Taken together, these results suggest that dendritic NMDA spikes are also critically important for cellular mechanisms of perception.

### Hippocampus: Perception of Space

Several lines of evidence have shown that the perception of the location of an animal in the external environment is one of the most common brain functions (Moser et al., 2008). In the hippocampus, spatial information is likely encoded by the firing of dendritic spikes in CA1 pyramidal neurons. In this type of neuron, dendritic Ca^2+^ spikes (Kamondi et al., 1998) or NMDA-dependent Ca^2+^ spikes (Grienberger et al., 2014) contribute to the generation of complex spikes. Moreover, place cells fire complex spikes when animals are in the place field, as shown in studies using *in vivo* whole-cell techniques with freely moving mice (Epsztein et al., 2011) and head-restricted mice in a virtual-reality environment (Harvey et al., 2009). More recently, a two-photon imaging study in behaving mice (Sheffield and Dombeck, 2015) has directly shown the contribution of dendritic Ca^2+^ spikes to the representation of the place field in the dendrites of CA1 neurons. In addition to these studies, dendritic spikes induced by the integration of two different inputs from the entorhinal cortex and hippocampal CA3 region have been shown to increase both the place field firing rates and the appearance of complex spikes in CA1 pyramidal neurons (Bittner et al., 2015).

## Conclusion

It has been postulated that the so-called “executive control system” is essential for perception, and that top-down control from this system that is responsible for higher brain functions modulates the activities of the primary sensory cortex in order to refine and define the sensory signal (Gilbert and Sigman, 2007; Gilbert and Burgess, 2008). This hypothesis seems to implicitly assume that there are specific brain regions dedicated to executing higher brain functions, and that the trains of impulses sent out along the axons of the neurons responsible for the executive control system constitute the basis for neuronal correlates of perception. In humans, the prefrontal cortex is thought to be responsible for the executive control system.

Results of the recent *in vivo* studies summarized in this review article indicate strong physiological relevance of dendritic spike generation in perception, and suggest that dendritic Ca^2+^ spikes and NMDA spikes in the somatosensory and hippocampal cortex support not only neuronal plasticity, but also the computational aspect of neuronal activities. In S1 pyramidal cells, TCI from M2 evokes dendritic spikes and somatic firing (Manita et al., 2015), which constitutes the late component, a necessary condition for sensory perception. These findings imply that the activities of neurons in the primary sensory cortex are not secondary to the executive control system, but rather that they constitute an integral part of the executive control system. We hypothesize that dendritic Ca^2+^ spikes and NMDA spikes in the pyramidal cells of primary cortices are not only necessary but may also be sufficient for sensory perception. This may be the case for other brain functions as well since the axonal pattern of innervation, similar to the one from M2 to S1, is ubiquitous across the cerebrum (Rockland and Virga, 1989; Coogan and Burkhalter, 1990; Cauller et al., 1998). We propose that dendritic spikes occurring in various regions of the brain, including S1, are the basis of cognitive functions such as perception, motor planning, attention and even self-awareness.

Although dendritic activities in the primary sensory cortex may indicate the occurrence of sensory perception, the contents of the perceived information might be represented separately in a brain region other than the primary cortex, and the outputs from the primary cortex might need to be conveyed to those systems for the sensory information to be recognized. One of the most important questions that should be addressed in future studies is whether dendritic spikes in the primary sensory cortex are associated with mental representation of perception, or whether there are regions responsible for perception located elsewhere, downstream of the primary sensory cortex. Recent technical advances allow researchers to study neuronal activities during behavioral tasks, and are beginning to provide clues to elucidate the mechanisms underlying the mind–body problem. To further our understanding of this issue, we need to study the dynamic coordination of neuronal activities, particularly dendritic activities, of populations of various neuron types, distributed across various parts of the brain.

## Author Contributions

All authors wrote the initial draft of the manuscript, and critically reviewed the manuscript. The final version of the manuscript was approved by all authors.

## Conflict of Interest Statement

The authors declare that the research was conducted in the absence of any commercial or financial relationships that could be construed as a potential conflict of interest.
